# Deleterious alleles in the context of domestication, inbreeding, and selection

**DOI:** 10.1111/eva.12691

**Published:** 2018-09-08

**Authors:** Mirte Bosse, Hendrik‐Jan Megens, Martijn F. L. Derks, Ángeles M. R. de Cara, Martien A. M. Groenen

**Affiliations:** ^1^ Animal Breeding and Genomics Wageningen University & Research Wageningen The Netherlands; ^2^ Centre d’Ecologie Fonctionnelle et Evolutive CNRS Université de Montpellier Université Paul Valéry Montpellier 3 EPHE, IRD Montpellier France

**Keywords:** deleterious alleles, domestication, genetic load, inbreeding, selection

## Abstract

Each individual has a certain number of harmful mutations in its genome. These mutations can lower the fitness of the individual carrying them, dependent on their dominance and selection coefficient. Effective population size, selection, and admixture are known to affect the occurrence of such mutations in a population. The relative roles of demography and selection are a key in understanding the process of adaptation. These are factors that are potentially influenced and confounded in domestic animals. Here, we hypothesize that the series of events of bottlenecks, introgression, and strong artificial selection associated with domestication increased mutational load in domestic species. Yet, mutational load is hard to quantify, so there are very few studies available revealing the relevance of evolutionary processes. The precise role of artificial selection, bottlenecks, and introgression in further increasing the load of deleterious variants in animals in breeding and conservation programmes remains unclear. In this paper, we review the effects of domestication and selection on mutational load in domestic species. Moreover, we test some hypotheses on higher mutational load due to domestication and selective sweeps using sequence data from commercial pig and chicken lines. Overall, we argue that domestication by itself is not a prerequisite for genetic erosion, indicating that fitness potential does not need to decline. Rather, mutational load in domestic species can be influenced by many factors, but consistent or strong trends are not yet clear. However, methods emerging from molecular genetics allow discrimination of hypotheses about the determinants of mutational load, such as effective population size, inbreeding, and selection, in domestic systems. These findings make us rethink the effect of our current breeding schemes on fitness of populations.

## 1 GENETIC LOAD AND INBREEDING

**Figure 1:**
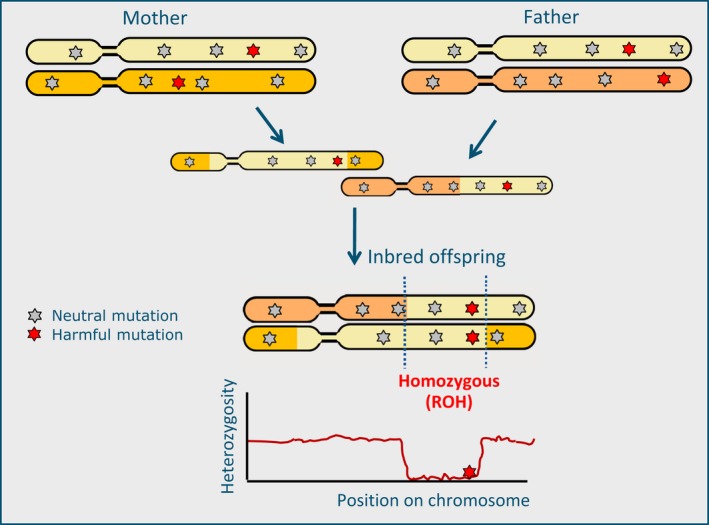
Genomic consequences of inbreeding. When parents are related, two identical haplotypes can be passed on to their offspring that are identical by descent. Therefore, no genetic variation exists between the two inherited copies in the inbred offspring. Such homozygous stretches (ROH: Runs Of Homozygosity) can be seen as long homozygous regions without polymorphisms in individual genomes. In ROH, harmful mutations have a higher chance of becoming homozygous and being expressed because the haplotypes carrying the harmful mutation stem from a common ancestor

Each genome carries deleterious mutations that can potentially affect fitness and health. According to population genetics theory, this mutational load depends on multiple factors such as mutation rate, demographic history, and selection. Most deleterious mutations are (at least partly) recessive, implying that their harmful nature will only be exposed in homozygous state. Since strongly detrimental variants are at low frequency (Mukai, Chigusa, Crow, & Mettler, 1972), homozygosity of these variants is most likely to occur due to inbreeding. Inbreeding is the inheritance of identical copies of genetic material from related parents and causes long homozygous regions in the genome of the offspring (ROH: Runs Of Homozygosity, see Figure 1; Curik, Ferencakovic, & Solkner, 2014). The potential negative impact that inbreeding will have on health and reproduction compared to an outbred population is referred to as “*genetic load*” (Crow, 1970a,b), which is mainly caused by the expression of recessive homozygous harmful mutations (Garcia‐Dorado, 2003; Lynch, Conery, & Burger, 1995).

### 1.1 Inbreeding depression

Small populations are more likely to suffer from inbreeding, which can negatively impact health and reproduction (Lynch et al., 1995). The decline in fitness observed in inbred progeny, relative to outbred progeny, is known as “*inbreeding depression*” (Keller & Waller, 2002). Inbreeding depression has largely been attributed to the accumulation of recessive harmful mutations in the genome: Inbreeding increases the probability of these mutations to become homozygous (i.e., Agrawal & Whitlock, 2012; Charlesworth & Willis, 2009; Ohta, 1973). Apart from increased homozygosity of recessives, the overdominance hypothesis assumes a heterozygote advantage, and therefore, overall loss of diversity due to inbreeding reduces the advantage of the heterozygotes (Crow, 1970a,b). In this paper, we focus on the detrimental effect of mostly recessive deleterious alleles, and their combined effect referred to as the mutational load.

With advancing sequencing technologies, the mutational load in the genome of an individual can be estimated from sequence data with increasing accuracy. We should bear in mind that the number of deleterious mutations does not necessarily need to differ between an inbred and an outbred individual for differences in fitness to exist; the key concept here is that while harmful mutations generally have a small fitness effect in heterozygous state, in homozygous state, they are expressed, causing, for instance, heritable diseases (Figure 1). Since ROH are formed because both haplotypes are identical by descent (IBD), the probability of a recessive deleterious allele with a low‐frequency *p* to become homozygous is higher in such regions than outside IBD regions (frequency *p* ^2^: (Szpiech et al., 2013).

### 1.2 Identification of deleterious alleles

Alleles with a putative effect on the phenotype, both beneficial and deleterious, are thought to be younger than variants with no effect with the same allele frequency (Maruyama, 1974). Understanding the factors that cause harmful mutations to increase in frequency in a genome will facilitate prediction of genetic load in current populations, but also aid to avoid a high genetic load in the future (Garcia‐Dorado, 2012). A popular approach to identify harmful mutations that are (almost) lethal is to screen populations for the absence of specific variants in homozygous state. If populations contain heterozygous carriers of such variants, homozygotes would be expected based on allele frequencies and carrier–carrier matings (Derks et al., 2017; Pausch et al., 2015; VanRaden, Olson, Null, & Hutchison, 2011). This method relies on the biological implications of the lethality of a variant—resulting in an absence of the allele in homozygous state. Depending on the frequency of the lethal allele in the population, large sample size is often required to identify such alleles (Derks et al., 2017). Therefore, most of the studies implementing this depletion of homozygotes approach use large panels of genotyped animals. An alternative approach is to predict deleteriousness from the functionality of a mutation. Next‐generation sequencing has opened up exciting possibilities to actually pinpoint potentially harmful mutations in individual genomes (Henn, Botigue, Bustamante, Clark, & Gravel, 2015; Li et al., 2010). The deleteriousness of a variant can be predicted based on its effect on gene functioning (such as protein changing, stop‐gain, stop‐lost), for example by assessing the degree of conservation of an amino acid residue across species (Cooper et al., 2005; Kumar, Henikoff, & Ng, 2009; Wang, Li, & Hakonarson, 2010). The implementation of multiple genome annotations, such as specific gene function or regulatory elements, has proven successful for in silico predictions of the effect of disease causing mutations in humans (Kircher, Witten, Jain, B. J. O'Roak, & Shendure, 2014). These techniques have recently been applied to estimate genetic load in human populations and domesticated species (Charlier et al., 2016). However, we need to remain cautious with such assessments of deleteriousness, since they rely on predictions that mostly have not been validated with experiments. High‐impact variation may very well not be lethal in a domestic setting and could even be perceived beneficial if they serve a particular breeding goal. Nevertheless, such advances in predicting the effect of variants have high potential for bridging the gap between sequence information and fitness effects.

### 1.3 Application to domestic species

A combination of both approaches to detect deleterious variants has proven successful in cattle (Pausch et al., 2015). Recent findings based on predicted deleterious variants from sequence data corroborate the classical theory by Mukai et al. (1972) that deleterious alleles are generally at low frequency (e.g., Mezmouk & Ross‐Ibarra, 2014). Multiple factors contribute to inbreeding depression, and epistatic effects across loci should be considered as well. Nevertheless, quantifying harmful mutations in single genomes is an important first step toward the “genomic characterization of genetic load.” An increase in studies assess mutational load in sequence data from domestic species. Some general trends about the effects of domestication on the burden of harmful mutations are now emerging (Makino et al., 2018; Zhou, Massonnet, Sanjak, Cantu, & Gaut, 2017). Here, we review recent studies on mutational load in domestic species and use re‐sequence data from pig and chicken to demonstrate how specific hypotheses about the burden of deleterious variants can be tested. Specifically, we will discuss two major drivers of mutational load in genomes: bottlenecks and selection.

## 2 THE DOMESTICATION BOTTLENECK

Domestication of plants and animals has a major impact on the domesticated species in terms of effective population size and selection pressure. This in turn could negatively affect the mutational load and cause genetic erosion (diminishing gene pool). In the context of inbreeding, lethal variants will quickly be purged from small populations (Charlier et al., 2016), but the frequency of slightly deleterious mutations is expected to rise as natural selection is less effective (Kimura, 1963). Past population bottlenecks have been proposed to drive mutational load in human populations (Li et al., 2010; Lynch et al., 2016), but the nature and strength of the impact is still debated (Henn et al., 2015; Lohmueller, 2014; Simons & Sella, 2016; Simons, Turchin, Pritchard, & Sella, 2014).

### 2.1 Domestication increases load

Several studies report the effect of domestication on mutational load. In line with predictions, the bottlenecks associated with domestication have not only reduced genetic diversity of domestic species, but also increased the mutational load in them as well. Domestication bottlenecks have indeed been suggested to have substantially increased the mutational load in dogs, referred to as the “legacy of domestication” (Cruz, Vila, & Webster, 2008). In dog genomes, the ratio of amino acid changing heterozygosity to silent heterozygosity (variants presumed to have no effect) was higher than in their wild ancestors, gray wolves (Marsden et al., 2016). These results indicate that the ability of purifying selection to remove weakly deleterious variants is lowered by the bottlenecks. A similar phenomenon has been reported for domestic horse genomes, where an excess of deleterious mutations is thought to be caused by domestication and inbreeding (Schubert et al., 2014). Also in crops, deleterious mutations seem to have accumulated in domestic lineages (Kono et al., 2016; Lu et al., 2006).

### 2.2 Type of bottleneck is important

The time frame in which the bottleneck occurs has a strong effect on the potential of natural selection to eliminate harmful mutations. If the bottleneck is severe and sudden, (local) genomic recombination is scarce, then drift can exert a maximal effect. If, however, the same population would decline over a long period of time, purging might enable deleterious variants to be removed from the population, resulting in a lower load (Charlier et al., 2016; Hedrick & Garcia‐Dorado, 2016). This begs the question of whether it was domestication itself that increased mutational load, or rather one of the processes that co‐occurred with it. The strong reduction in effective population size, going from wild to domesticated, could have driven the deleterious alleles to higher frequency (Liu, Zhou, Morrell, & Gaut, 2017). Artificial selection could have reduced the effective population size even further.

Most domestic animal species are thought to have experienced a relatively strong population bottleneck, although the picture can become complex for species that went through multiple domestications in different regions. In livestock, domestication is no longer seen as a single, discrete event. Rather, substantial and continuous gene flow from wild populations has occurred during the process of domestication (Frantz et al., 2015; Scheu et al., 2015). In rice, Liu and colleagues suggest that it was not domestication itself, but the shift in mating system from outcrossing to predominantly selfing that had a substantial influence on mutational load (Liu et al., 2017). This suggests that the process of domestication as well as the management regime under which current lines have been formed could have influenced the occurrence of deleterious mutations in domestics. The intensity of the domestication bottleneck is thought to have influenced the difference in mutational load between annual and perennial crops (Zhou et al., 2017). A recent analysis on multiple domesticated species concludes that in domestic plant and animal genomes, an elevated proportion of deleterious genetic variation is present, with European pigs as an exception (Makino et al., 2018). This creates opportunity to investigate further how domestication has elevated mutational load.

### 2.3 Case study in pig and chicken: application to sequence data

We used genotype and re‐sequence data from pigs and chicken to investigate the mutational load in wild and domestic populations, as described in supplementary materials. These species represent highly different domestication histories and selection regimes. We compared the ratio of predicted deleterious heterozygosity with silent heterozygosity within individual genomes to estimate mutational load in domestic and (semi) wild pigs and chickens, using the method described by Renaut and Rieseberg (2015), applied to sunflowers.

Pigs were domesticated at least twice, independently, giving rise to the current Asian and European‐based domestic clades (Kijas & Andersson, 2001; Larson et al., 2005). European and Asian wild boar form an excellent model for their wild ancestors, enabling direct comparisons between the wild and domestic form (Bosse, Megens, Frantz, et al., 2014; Bosse, Megens, Madsen, et al., 2014). The use of pigs from two different geographic regions (Asia and Europe) and subjected to different domestication events (wild and domestic, including local and commercial populations) enables the study of the impact of demography and domestication on the distribution of deleterious mutations. In chicken, however, the wide variety of domestic breeds are thought to stem from red jungle fowl (Fumihito et al., 1996) and involve more complex demographic history, including multiple regional centers of domestication across Asia (Miao et al., 2013).

#### 2.3.1 Case study: increased load is context‐specific

**Figure 2:**
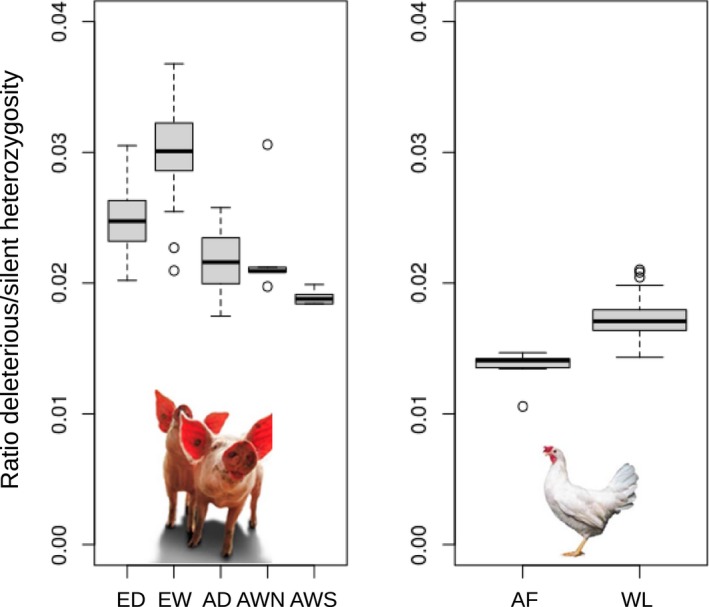
Mutational load in *Sus scrofa* and *Gallus gallus*. Mutational load in individual genomes, calculated as the ratio of predicted deleterious heterozygous sites over synonymous heterozygous sites. (a) Pigs. ED = European domestic; EW = European wild; AD = Asian domestic; AWN = North Asian wild; AWS = South Asian wild. (b) chicken AF = African village chicken, putatively evolutionary closer to red jungle fowl. WL = commercial white layer lines

The estimated mutational load in commercial chicken lines is higher than in African village chicken, both from estimates based on heterozygous variants and from estimates from homozygous variants (Figure 2b, Supporting Information Figure S1). An elevated mutational load in commercial chicken is corroborated by the analysis of pooled chicken data in Makino et al. (2018).

The estimated mutational load was generally higher in European pig genomes than in Asian pigs (Figure 2a). So far, these analyses support the general view that domestication coincides with a population bottleneck that underlies the increase in load in the domestic form, which was also found by Makino et al. (2018). Studies in human populations also indicate that individuals from populations that have experienced bottlenecks tend to carry more deleterious alleles (Fu, Gittelman, Bamshad, & Akey, 2014; Lohmueller et al., 2008). This is in agreement with the pattern of deleterious mutation observed in pigs, where European populations had a higher proportion of deleterious variants. Rather than affected by domestication, this seems to be related to the demography of the ancestral, wild boar population. In European wild boar, population bottlenecks during the Last Glacial Maximum were more severe than in Asia, which largely explains the lower genetic diversity of European wild boar (Groenen et al., 2012). Thus, the higher number of deleterious variants observed in European pig populations, especially in wild boar, reflects the typically negative correlation between genetic diversity and the incidence of deleterious mutations.

By contrast, the European commercial pig genomes contained a lower ratio of deleterious over silent heterozygosity than European wild boar. This finding suggests that the mutational load is lower in the domestic lineage (Figure 2a), corroborating the study by Makino et al. (2018). As European commercial pigs have a higher genetic diversity because of gene flow from Asia during the Industrial revolution (Bosse, Megens, Frantz, et al., 2014; Bosse, Megens, Madsen, et al., 2014; White, 2011), this influx could have reduced the mutational load in domestic pig genomes as well. Alternatively, the recent bottlenecks within European wild boar populations could have relaxed purifying selection on slightly deleterious variants in Europe and increased mutational load. However, for dogs, recent inbreeding was insufficient to explain the observed accumulation of deleterious alleles, suggesting an older cause (Marsden et al., 2016). Herrero‐Medrano et al. (2014) suggested that European local pig breeds have exceptionally high levels of both ROHs and potentially damaging mutations, probably a combination of their European heritage and current low effective population size.

The absolute number of homozygous deleterious variants inferred from the genomes of the different pig groups did not differ much, but the ratio of homozygous deleterious over homozygous neutral variants was higher in European pig and wild boar compared to Asian pigs (Supporting Information Figure S1). Variants with a strong effect are thought to be younger than variants with no effect with the same allele frequency. Therefore, the majority of deleterious variants are thought to be derived alleles, which were also found by Makino et al. (2018). When comparing breeds or populations with a relatively deep phylogenetic split, polarizing alleles using an outgroup is recommended since the distance to the reference genome could introduce a bias (Lohmueller, 2014; Makino et al., 2018; Simons & Sella, 2016).

### 2.4 Consequences of increased load

Whether the high estimated mutational load in domestic genomes will lead to lowered viability is questionable. Many commercial lines are bred especially for reproduction‐related traits or through the production of hybrids, which will dilute the effect of mutational load. Current ongoing work has shown that life history traits such as lifespan or propagule size are a key to understanding levels of genetic diversity and mutational load (Romiguier et al., 2014). So far, the results on mutational load estimated from the ratio of synonymous to nonsynonymous mutations are fairly evident in indicating that purifying selection is higher in the wild (Chen, Glemin, & Lascoux, 2017). The sources of genetic variation that create genetic diversity where selection can act upon are the same for both domestic and wild animals. However, in most circumstances, domestic animals have been so strongly selected for the sake of production, that their genetic diversity is extremely low. Moreover, artificial selection for specific traits sometimes results in so much inbreeding that it is threatening the survival of the breed. Clear examples can be seen in dogs such as the Norwegian Lundehund suffering from intestinal problems, but cancer, eye, and heart diseases are also common (Kettunen, Daverdin, Helfjord, & Berg, 2017; Schoenebeck & Ostrander, 2014). Combined, these effects of inbreeding in domestics have led to animals and plants that may have lost their ability to face environmental challenges, should they come from climate change or new diseases. Finally, the circumstances in which domestic animals are kept do not reflect the (harsh) environment they face in the wild. The loss of adaptive potential, disease resilience, and fitness reduction is of major concern for monocultures such as bananas and in salmon (Araki, Berejikian, Ford, & Blouin, 2008; Garcia de Leaniz et al., 2007). Together with the fact that the traits selected in domestic animals are usually deleterious in the wild make the detrimental effects of the potentially harmful mutations hard to observe (Hedrick & Garcia‐Dorado, 2016).

## 3 ARTIFICIAL SELECTION AND MUTATIONAL LOAD

**Figure 3:**
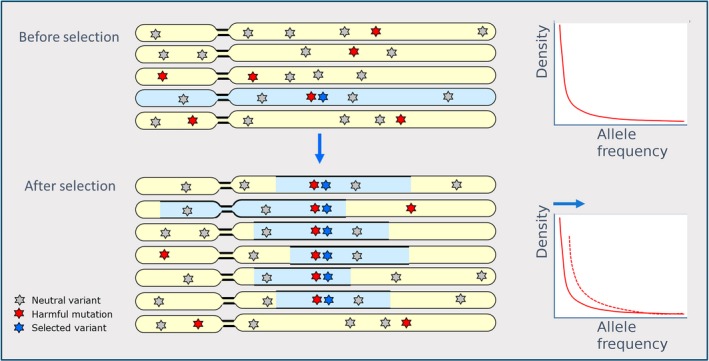
Genetic hitch‐hiking of deleterious alleles in selected regions. Under neutrality, deleterious alleles are present in a population at low frequency (indicated in red). If a deleterious allele lies on the same haplotype as a selected variant (indicated in blue), linkage is strong, and if the selective advantage s of the advantageous allele is stronger than the summed selection coefficient against all (slightly) deleterious alleles ∑*s*, the harmful mutations will rise in frequency despite their deleterious nature as demonstrated in the allele frequency spectrum in the population before – and after selection

The strong artificial selection that is associated with domestic populations can increase inbreeding in commercial lines, as was already mentioned by Lush (1946). Not only the reduced effective population size, therefore, but also the selection for favoured gene variants may have increased homozygosity within domestic animals. In addition to the increase in homozygosity due to drift effects, selection constraints on detrimental variants may be lifted if they are in LD with a favoured allele that is strongly selected for. (Maynard Smith & Haigh, 1974). If the selection coefficient against those mutations, combined, is lower than the selection coefficient of the preferred allele that lies on the same haplotype, the allele frequency of the deleterious variant(s) is expected to rise due to genetic hitch‐hiking. Therefore, mildly harmful mutations are thought to be over‐represented in regions of the genome under selection (Figure 3; Charlesworth, 2006; Good & Desai, 2014).

### 3.1 Increased load due to selection

In humans, deleterious alleles are thought to have increased in frequency due to linkage to sites that have been under positive selection. (Chun & Fay, 2011). Indeed, in domestic species, we find similar evidence of selected regions to be over‐represented with predicted deleterious alleles. In dogs, an increased load was found within regions under selection, inferred from the genome (Marsden et al., 2016). A different approach is to assess the mutational load in genes known to affect phenotypic traits of interest. In plants, such genes were shown to contain proportionally more deleterious variants (Kono et al., 2016; Lu et al., 2006; Mezmouk & Ross‐Ibarra, 2014). The linkage of deleterious variants to genes under balancing selection could also lead to a local overrepresentation of deleterious variants. A recent study in cattle (Kadri et al., 2014) showed that a long known antagonism between fertility and milk production is actually due to such a phenomenon. While a major QTL for fertility with effects in milk production is under balancing selection in nature, the change to directional selection in selection for milk production has led to an extremely reduced fertility in cows. Balancing selection in livestock has probably an underestimated role yet to be explored.

### 3.2 Case study in pig and chicken: load in runs of homozygosity

**Figure 4:**
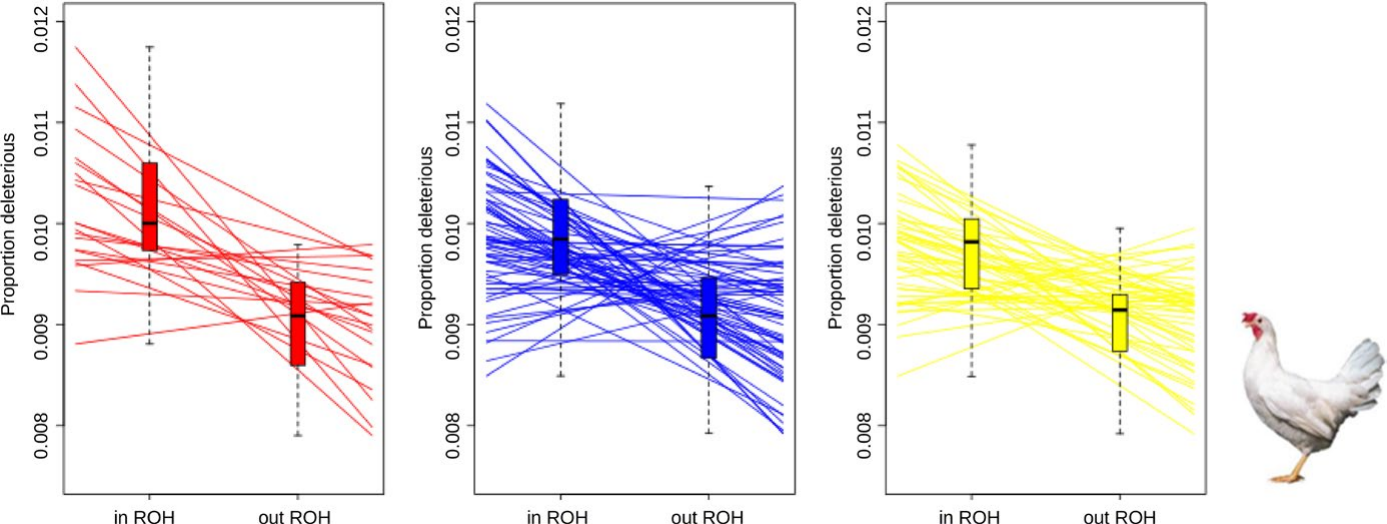
Deleterious alleles within and outside ROH in chicken. Proportion of deleterious homozygous variants within and outside ROH‐regions in three different white layer lines. Paired t test Line1 *p *= 6.65e‐05; Line2 *p *= 1.11e‐07; Line3 *p *= 5.684e‐07

**Figure 5:**
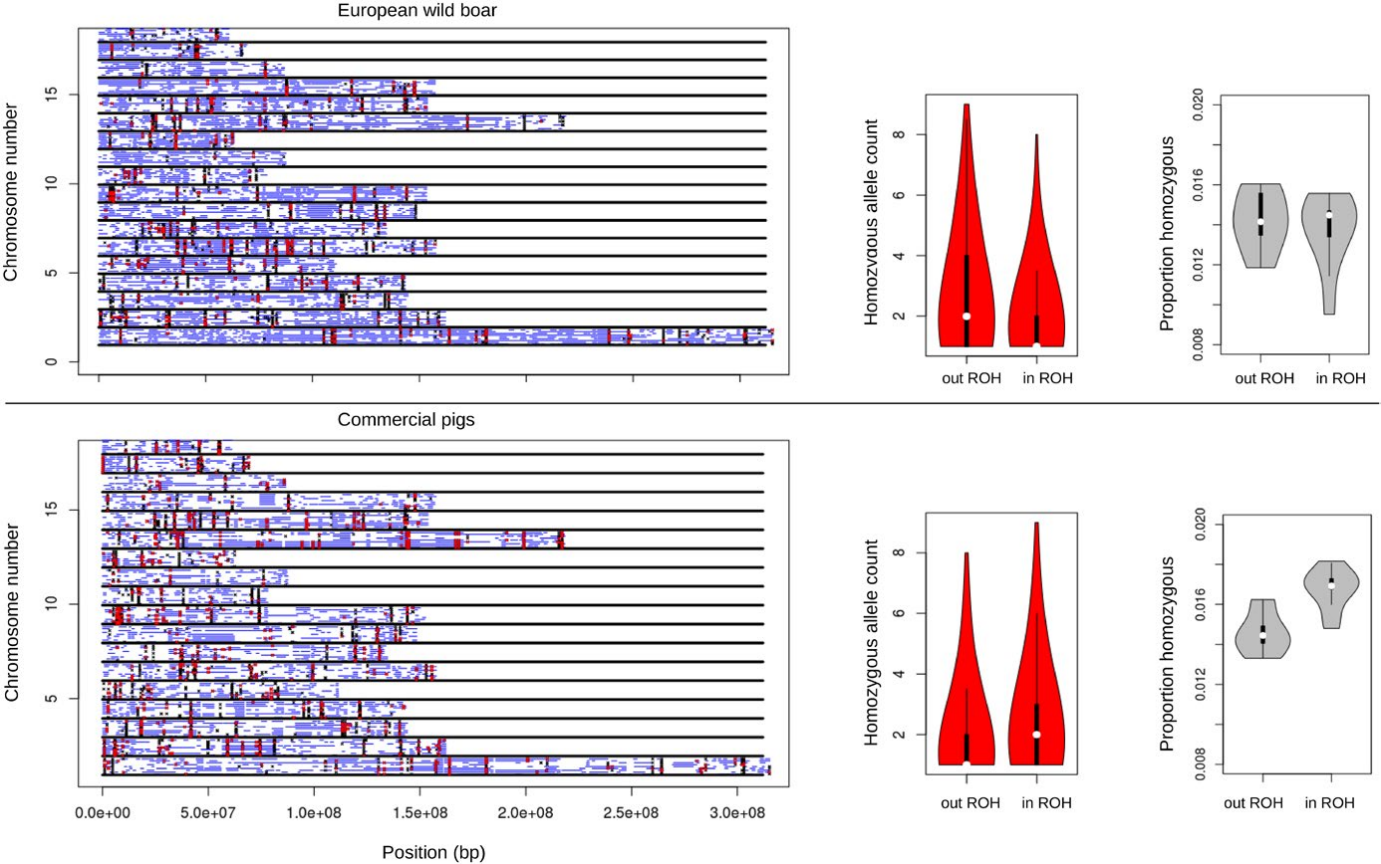
Distribution of deleterious alleles within the genomes of commercial pigs and wild boars. (a) The *x*‐axis represents length of the chromosome, and numbers on the *y*‐axis indicate chromosomes. Blue horizontal bars represent ROH within the genome of an individual, red crosses pinpoint the location of predicted deleterious variants within ROH, and black crosses mark the position of homozygous deleterious alleles outside ROH. (b) Violin plots summarizing the frequency of homozygous deleterious alleles within and outside ROH‐regions. (c) The proportion of homozygous deleterious alleles compared to neutral homozygous alleles within and outside ROH‐regions

Together, inbreeding and strong directional selection can increase the proportion of homozygous segments in individual genomes. Following the rationale of Szpiech et al. (2013) that ROH are enriched for deleterious variants (in homozygous state), we tested this hypothesis in pig and chicken lines. In three white layer lines, the number of predicted deleterious homozygous variants was higher in ROH‐regions compared to the rest of the genome. As a result, ROH contain proportionally more homozygous deleterious alleles than the rest of the genome (Figure 4), in line with findings in humans (Szpiech et al., 2013). Also, in commercial European pigs, ROH‐regions contained proportionally more deleterious homozygous variants. Interestingly, however, European wild boar did not display this pattern (Figure 5c).

### 3.3 Disentangling selection and drift

A higher proportion of deleterious alleles in ROH were also observed in cattle (Zhang, Guldbrandtsen, Bosse, Lund, & Sahana, 2015). In humans, the longest class of ROH was most enriched for deleterious variants in humans, whereas in cattle, shorter ROH contained proportionally more deleterious alleles (Zhang et al., 2015). A possible explanation for these different patterns is that in humans, inbreeding is mostly responsible for the formation of long ROH and hard selective sweeps are rare (Hernandez et al., 2011) By contrast, in cattle, artificial selection intensity is high and sweeps are abundant (Kim et al., 2013). This indicates that the different mechanisms that result in ROH, namely selection and drift, result in different patterns of detrimental alleles. In dog genomes, when regions under selection were excluded, an increase in deleterious alleles compared to wolves could still be observed, suggesting that drift rather than the hitch‐hiking effect associated with selection is causing the increased load in dogs (Marsden et al., 2016). Also, the role of recombination in generating genomic diversity patterns and ROH should not be neglected; recombination rate locally influences effective population size in a genome, which in turn affects the efficiency of natural selection (Begun & Aquadro, 1992; Bosse et al., 2012). Finally, the number of generations since the last common ancestor and the speed of inbreeding influence the length of ROH stemming from inbreeding, and the associated mutational load (Hedrick & Garcia‐Dorado, 2016). Therefore, minimizing inbreeding in modern breeding practices by specifically avoiding long IBD segments in optimal contributions may avoid an increase in mutational load (de Cara, Villanueva, Toro, & Fernandez, 2013a,b).

Distinguishing regions under selection and IBD segments due to inbreeding may shed new light on the assumed detrimental effects of domestication. Specifically targeting genes known to be associated with favorable traits, or regions of reduced heterozygosity on a population scale, can aid in distinguishing ROHs stemming from inbreeding from ROHs stemming from selection.

#### 3.3.1 Case study: deleterious allele frequency and ROH

As can be seen in Figure 5, predicted deleterious variants in ROH occur often in regions where ROH occur in the same genomic region in multiple individuals. These predicted harmful mutations seem to be at higher frequency than expected if they were homozygous primarily due to inbreeding, suggesting a strong role of hitch‐hiking through artificial selection. Such co‐occurrence of ROH in individuals is an indication of selection for a favorable haplotype in a population, suggesting that most deleterious alleles are maintained due to hitch‐hiking along with a selected variant at that locus. Indeed, the allele frequency of homozygous deleterious alleles in the population was higher in ROH‐regions for commercial pigs, whereas in European wild boar, the allele frequency of deleterious homozygous variants was higher outside ROH‐regions (Figure 5b). A likely explanation for this distinct pattern could be that past bottlenecks in European wild boar have resulted in fixation/elevation of many homozygous deleterious variants. Whereas, the increase in deleterious homozygous variants in commercial pigs is driven by more recent processes such as artificial selection.

## 4 CONCLUSIONS

Based on previous work as well as on our own analyses, we conclude that despite the strong artificial selection on commercial breeds, mutational load can be high. The probability of a deleterious allele to rise in frequency in a population depends not only on population size (drift effects), but also on its selection coefficient (Whitlock, 2000). An important factor to keep in mind is that if the population is not at mutation‐drift equilibrium, such as during rapid population growth, the distribution of deleterious variants is affected and load can increase rapidly (Casals et al., 2013; Gazave, Chang, Clark, & Keinan, 2013). The population bottleneck associated with domestication is thought to be generally long‐term and inbreeding increase slow, relative to breed formation which coincides with quick increase in inbreeding: both are relevant for domesticated breeds (Oldenbroek, 2017). A further issue is that selection acts differently in domestic animals, where the individuals who will contribute to leave offspring are chosen according to some trait that is being optimized. By artificially selecting these individuals, natural selection cannot act on the population as it does in the wild, and there is very little room for it to act (de Cara et al., 2013a,b). Moreover, “predicted to be deleterious” can actually signify “beneficial” in an artificial selection context. The prediction of deleteriousness relies on variants that are thought to have a high impact on the phenotype. Therefore, “deleterious” could mean “not generally tolerated in the wild,” but may be perfectly viable (or even highly viable and selected for) in a domesticated setting. Estimates of genetic load inferred from genomes are insensitive for the specific environmental circumstances of individuals carrying deleterious alleles, which may influence the impact of the predicted deleterious alleles on the fitness of an individual. We suggest some caution when inferring these general patterns of mutational load, since deleteriousness can be context‐dependent (Hedrick & Garcia‐Dorado, 2016).

### 4.1 Final remarks

Overall, domestication seems to have elevated load in most domestic species. However, domestication does not lead to genomes with high load per se, indicating that fitness does not need to decline. Accumulation of harmful mutations in the genome is facilitated by population bottlenecks and (artificial) selection, but the details of population demography and selection history are species‐ and breed specific. Some mechanisms seem to apply to multiple domestic species, such as an increase in load due to (domestication) bottleneck. Also, the ancestral load is an important factor influencing load in different breeds, since the source population determines which variants are present in the first place. Artificial selection may further increase load by the facilitation of hitch‐hiking of harmful mutations and further restrictions of effective population size. The rare example of lower load in European domestic pigs compared to European wild boars provides a more positive view on mutational load in domestics. In this case, either the process of domestication itself or the artificial selection and hybridization leading to current breeds may have lowered mutational load. We are only beginning to understand how different selection regimes have influenced the mutational load in domestic lineages, and how estimated mutational load and fitness effects are connected. Consistent or strong trends about the influence of domestication on mutational load are not yet clear. However, some common patterns are emerging. We have entered an exciting era in which population genetics theory can be tested with genomic data to shed new light on the interplay of demography, selection, mutational load and fitness. Integrating knowledge from humans, model species such as *Drosophila*, domesticated species such as livestock and crops, and endangered wild species in combination with in silico predictions will aid in understanding the genetic load across many different demographic and selection scenarios.

## 5 METHODS

### 5.1 Sampling

Re‐sequence data from a total of 76 pigs were retrieved from the European Nucleotide Archive (ENA) accession code ERP001813 and included 43 domestic pigs and 33 wild boars from Asia and Europe. Pigs were classified into five groups in accordance with their geographic origin and domestication status: Asian domestic pigs (AD; *N* = 16), North Asian wild boars (AWN; *N* = 5), South Asian wild boars (AWS; *N* = 5); European commercial pigs (ED; *N* = 27) and European wild boars (EW; *N* = 23). For chicken, re‐sequence data from eight African village chicken coming from different ecotypes in Kenya were obtained from (Ngeno, 2015). In addition, re‐sequence data from one commercial pig line (*N* = 9) and three chicken lines (*N* = 51;66;43) were obtained from (Derks et al., 2017; Derks, Megens et al. (submitted) and Derks et al., 2018) respectively.

^1^European Nucleotide Archive (ENA) https://www.ebi.ac.uk/ena/data/view/PRJEB1683; ^2^(Bosse et al., 2012) ^3^(Yang et al., 2017) ^4^(Derks et al., 2017) ^5^(Ngeno, 2015) ^6^(Ngeno et al., 2015) ^7^(Derks et al., 2018), ^8^(Derks, Megens et al. (submitted)).

### 5.2 Alignment and variant calling

All samples were sequenced with Illumina paired‐end short read sequencting technology (Illumina inc). Reads were trimmed to a phred quality >20 and minimum length of both pairs of 40 bp. For pigs, short read alignment was done against the Sus scrofa genome, build 10.2 (Groenen et al., 2012) using BWA‐mem. For chicken, reads were aligned to the chicken reference genome Galgal 4 (International Chicken Genome Sequencing 2004). Variants were called and filtered using GATK (Genome Analysis Toolkit 2.6‐4) on an individual basis to avoid biases resulting from unequal sampling across populations. INDEL realignment, base quality score recalibration, and UnifiedGenotyper module analyses were performed according to GATK Best Practices. These variants were further filtered for read depth between half and twice the average genome‐wide depth of that individual with a minimum depth of 4x. We only considered genic variants that had genotype quality scores >Q20.

### 5.3 Identification of potentially deleterious variants

To obtain the functional consequences of the variants for pigs, we used Variant Effect Predictor (VEP, (McLaren et al., 2016)) on the ENSEMBL Sus Scrofa 78gene set, 1‐to‐1 orthologues with cattle. For chicken, we used Variant Effect Predictor(VEP) v78 on the ENSEMBL Gallus gallus 78gene set. Variants within a gene with a missense annotation and variants labeled as synonymous variant were retained for downstream analyses. The deleterious effect of the missense mutations were predicted using SIFT (Kumar et al., 2009), and SIFT scores <0.01 were used as deleterious mutations in calculating the ratio deleterious/synonymous.

### 5.4 Deleterious variants and ROH

Runs of homozygosity (ROH) were estimated from genotype data from the same individuals, obtained from (Yang et al., 2017) and (Derks et al., 2017) for pigs and from (Derks et al., 2018) for chicken. SNP genotyping was performed on the species‐specific Illumina 60K iSelect Beadchip for both species (Ramos et al., 2009). A ROH was defined as a genomic region of at least 1 Mb with at least 20 SNPs supporting the homozygous state, using the –homozyg option in PLINK v.1.9. This length reflects roughly 1 cM and consanguinity 50 generations ago (Howrigan, Simonson, & Keller, 2011), which is sufficient inbreeding for the purpose of this study, yet feasible with the density of our SNP chips. Number of deleterious and tolerated nonreference homozygotes overlapping and nonoverlapping ROH (Del‐ROH and Del‐non‐ROH, respectively) were counted separately in each individual. Differences between the fraction of deleterious and tolerated variants in ROH per individual were tested for significance using the paired t test as implemented in R.

## DATA ARCHIVING STATEMENT

Re‐sequence data from a total of 76 pigs were retrieved from the European Nucleotide Archive (ENA) accession code ERP001813. Re‐sequence data from eight African village chicken were obtained from (Ngeno, 2015 and described in Ngeno et al., 2015). Since Ngeno, 2015 is a PhD thesis, the relevant vcf files of the village chicken are deposited into https://www.animalgenome.org/repository/pub/WUR2018.0809/. In addition, re‐sequence data from one commercial pig line (*N* = 9) and three chicken lines (*N* = 51;66;43) were obtained from (Derks, Megens et al. (under review), Derks et al., 2018), respectively. Since (Derks, Megens et al. (under review) is not accessible yet, the relevant vcf files are deposited into https://www.animalgenome.org/repository/pub/WUR2018.0809/. Genotype data from the same individuals were obtained from (Yang et al., 2017) and (Derks et al., 2017) for pigs and from (Derks et al., 2018) for chicken. The data obtained from Derks et al. (2018) was restricted but will be made available upon specific request to ensure repeatability is possible.
