# Supplementary figures and images for: Deleterious alleles in the context of domestication, inbreeding, and selection
Source: Evol Appl. 2018 Sep 8;12(1):6–17. doi: 10.1111/eva.12691 (PMC6304688; doi:10.1111/eva.12691)

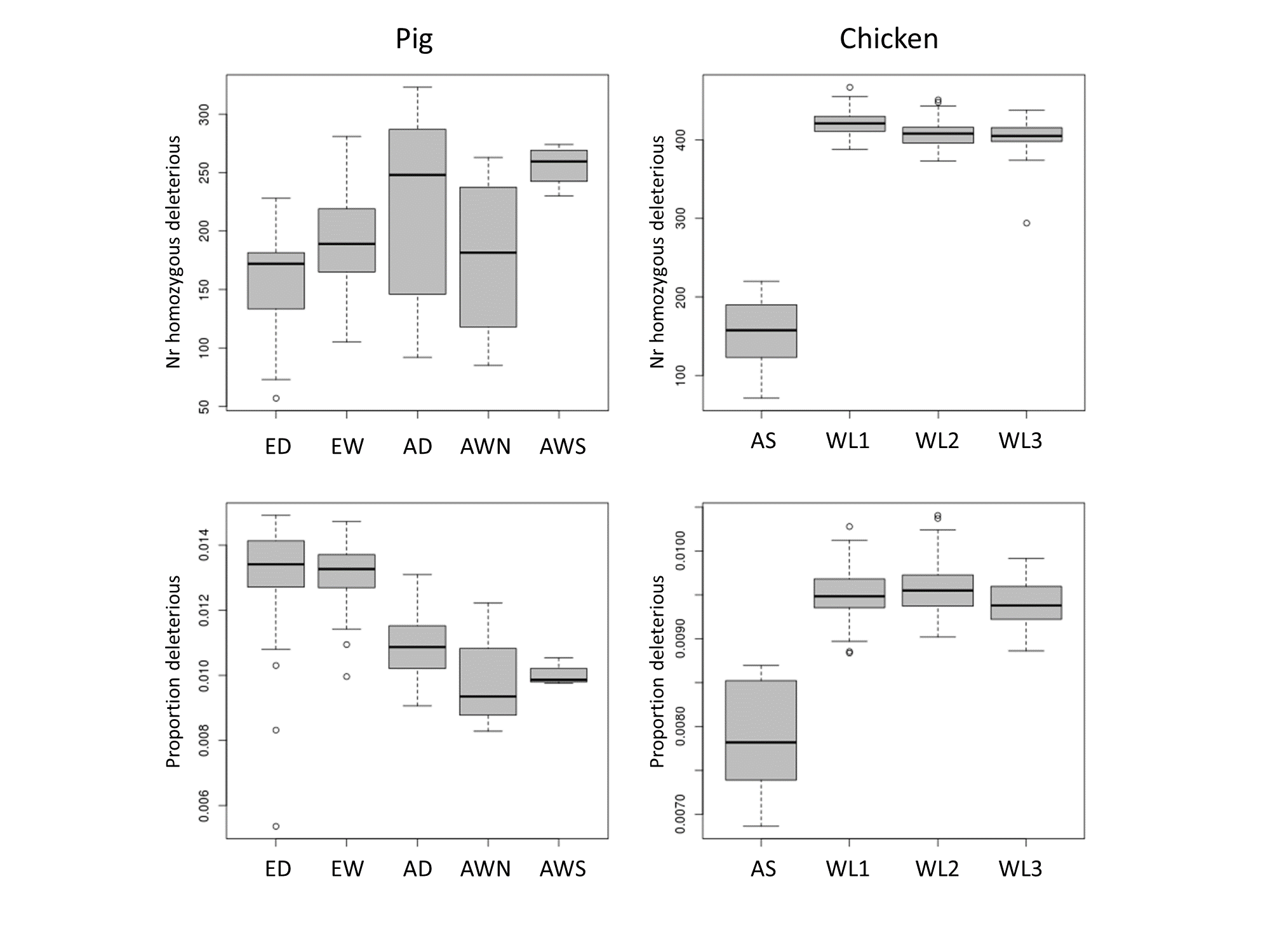

Supplement: Supplementary file 1 [file EVA-12-6-s001.png]
